# Pathologic Bladder Microenvironment Attenuates Smooth Muscle Differentiation of Skin Derived Precursor Cells: Implications for Tissue Regeneration

**DOI:** 10.1371/journal.pone.0059413

**Published:** 2013-04-01

**Authors:** Cornelia Tolg, Alya Ahsan, Shaalee Dworski, Tyler Kirwan, Jeffery Yu, Karen Aitken, Darius Jehan Bägli

**Affiliations:** 1 Developmental and Stem Cell Biology, Research Institute, Toronto, Ontario, Canada; 2 Division of Urology, Hospital for Sick Children, Research Institute, Toronto, Ontario, Canada; 3 Institute of Medical Sciences, University of Toronto, Toronto, Ontario, Canada; 4 Departments of Surgery & Physiology, University of Toronto, Toronto, Ontario, Canada; National Cancer Institute, United States of America

## Abstract

Smooth muscle cell containing organs (bladder, heart, blood vessels) are damaged by a variety of pathological conditions necessitating surgery or organ replacement. Currently, regeneration of contractile tissues is hampered by lack of functional smooth muscle cells. Multipotent skin derived progenitor cells (SKPs) can easily be isolated from adult skin and can be differentiated *in vitro* into contractile smooth muscle cells by exposure to FBS. Here we demonstrate an inhibitory effect of a pathologic contractile organ microenvironment on smooth muscle cell differentiation of SKPs. *In vivo*, urinary bladder strain induces microenvironmental changes leading to de-differentiation of fully differentiated bladder smooth muscle cells. Co-culture of SKPs with organoids isolated from *ex vivo* stretched bladders or exposure of SKPs to diffusible factors released by stretched bladders (e.g. bFGF) suppresses expression of smooth muscle markers (alpha SMactin, calponin, myocardin, myosin heavy chain) as demonstrated by qPCR and immunofluorescent staining. Rapamycin, an inhibitor of mTOR signalling, previously observed to prevent bladder strain induced de-differentiation of fully differentiated smooth muscle cells *in vitro*, inhibits FBS-induced smooth muscle cell differentiation of undifferentiated SKPs. These results suggest that intended precursor cell differentiation may be paradoxically suppressed by the disease context for which regeneration may be required. Organ-specific microenvironment contexts, particularly prevailing disease, may play a significant role in modulating or attenuating an intended stem cell phenotypic fate, possibly explaining the variable and inefficient differentiation of stem cell constructs in *in vivo* settings. These observations must be considered in drafting any regeneration strategies.

## Introduction

Bladder outlet obstruction, the result of congenital or acquired abnormalities such as posterior urethral valves, spina bifida, prostate hypertrophy, or neurogenic bladder leads to increased bladder pressure that over time induces bladder wall thickening and loss of bladder function. Treatment options for obstruction induced loss of bladder function are external urinary drainage or surgical bladder augmentation with gastrointestinal tissue segments. The specific physiology of gastrointestinal tissue, which is specialized for uptake of nutrients, results in complications such as acidosis and bacteriuria and possibly leads to an increased bladder cancer risk [1] [2]. An alternative, more physiologic tissue source to replace damaged bladder muscle is therefore highly desired. In recent years progress has been made toward the use of autologous, patient derived bladder cells in bladder tissue engineering or regeneration approaches [3]. For example, bladder smooth muscle cells (SMC) and urothelial cells have been isolated from bladder biopsies and expanded in culture. These cells were then used to seed scaffolds, creating engineered bladder tissue for augmentation surgery [3]. However, this expansion strategy may be counterintuitive as native bladder muscle cells continue to exhibit fixed phenopathology [4], [5].

Pluripotent progenitor cells are an alternative to the use of differentiated bladder cells. These cells can be isolated from several tissues and can then be differentiated into bladder cells [6]. For example, bone marrow mesenchymal stem cells express similar contractile proteins as bladder SMC [7] and have been differentiated into SMC *in vitro* by TGFbeta treatment or co-culture with urothelial cells [8], [9], [10]. In contrast, *in vivo*, bone marrow-derived mesenchymal stem cells preferentially differentiate into urothelial cells [11], [12]. Although these studies demonstrate that bone marrow stem cells are a potential source for bladder cells, the *in vitro* vs. *in vivo* context into which they are placed has profound effects on their programming. Moreover, harvest of these cells requires general anaesthesia, thereby limiting their use for tissue engineering.

More accessible alternatives to bone marrow stem cells are adipose tissue or skin derived progenitor cells (SKPs). Transplantation studies using acellular matrices seeded with adipose tissue derived stem cells appeared to improve bladder *in vivo* regeneration but contribution of progenitor cells to the final differentiated muscle cell population is still controversial [13], [14]. Recently, pluripotent progenitor cells from adult rodent and human skin have been isolated [15], [16]. These SKPs reside in the hair follicle niche and share characteristics with neural crest cells [17], [18], [19], [20]. While culture of SKPs as non-adherent spheres in EGF and FGF containing medium preserves their multipotent, undifferentiated state for several cell generations, growth factor withdrawal under adequate culture conditions induces differentiation into adipocytes, glia, neurons, chrondrocytes, and vascular SMC [16], [21]. *In vivo*, SKPs are currently being tested in models of spinal cord injury, bone injury, and wound repair [20], [22], [23], [24], [25], [26]. Nevertheless, despite these encouraging results, it still appears that *in vivo* differentiation of progenitor cells is often very inefficient. We postulate that the complexities of the various *in vivo* microenvironments are likely responsible for the observed inefficient *in vivo* differentiation of progenitor cells, and understanding their mechanistic basis may be critical to successful incorporation of progenitor cells into tissue renewal strategies.

At the cellular level, increased bladder pressure induces SMC proliferation and loss of differentiation. These changes in bladder SMC biology are the result of a modified, pathologic microenvironment. For example, bladder strain induces matrix metalloproteinase (MMP) 7 production and collagen degradation, ultimately resulting in release of activated EGF and stimulation of the EGF signalling pathway in bladder SMC [27], [28]. SMC transcriptome analysis identified mTOR as one cell signalling pathway that was activated by mechanical strain, hypoxia and denatured collagen, three convergent stimuli involved in bladder response to obstruction [29]. Moreover, recent protein-protein interaction analyses suggest that mTOR interacts closely with the epigenetic methyltransferase machinery (unpublished observations), which may represent a further mechanism by which progenitor cells respond to natural or engineered microenvironments. Rapamycin, a commonly used mTOR inhibitor, inhibits visceral SMC MMP production, cell proliferation, as well as de-differentiation *in vitro* [29]. The use of progenitor cells in bladder tissue regeneration requires progenitor cell re-programming and differentiation within the discrete context of strain-induced microenvironments.

In this study we assessed the effect of strain-exposed bladder cells on the differentiation of SKPs into SMC and tested whether inhibition of the mTOR pathway could be used to increase SMC differentiation of undifferentiated SKPs, similar to its ability to rescue de-differentiation in mature SMC. We observed a paradoxical response in SKPs to both strain-induced microenvironmental cues as well as to mTOR inhibition, in contrast to the responses of mature differentiated SMC.

## Materials and Methods

### Ethics Statement

All animal experiments were approved by the Hospital for Sick Children animal use committee following Canadian Council on Animal Care guidelines (protocol # 19076 Identification of Novel Treatment Strategies for Obstructive Bladder Disease in a Rat Model).

### Isolation of Skin Derived Progenitor Cells

SKPs were isolated from 6 weeks old female Sprague Dawley rats as described previously [20], [25]. In brief, rats were euthanized by CO_2_ inhalation. Back skin was depilated using wax strips and a piece of skin was removed. Skin was washed in cold HBSS (Invitrogen Life Technologies Inc., Burlington, ON) and fat layers were removed from the skin underside using forceps. Skin was then cut into small pieces using razor blades and digested with collagenase XI (1 mg/ml) (Sigma-Aldrich, St.Louis, MO)/HBSS for 1 hr at 37°C. Collagenase/skin cell suspension was passed through a 70 µm mesh to remove remaining tissue pieces. Collagenase/cell suspension was diluted with DMEM+Glutamax (Invitrogen Life Technologies Inc., Burlington, ON) and cells were isolated by centrifugation (5 min, 1200 rpm). Cells were plated in DMEM/F12 3∶1+ Glutamax medium (Invitrogen Life Technologies Inc., Burlington, ON) containing 2% B27 (Invitrogen Life Technologies Inc., Burlington, ON), 20 ng/ml EGF (PeproTech Canada Inc., Rocky Hill, NJ), 40 ng/ml FGF2 (PeproTech Canada Inc., Rocky Hill, NJ), Penicillin/Streptomycin (Lonza, Basel, Switzerland) and Fungizone (Invitrogen Life Technologies Inc., Burlington, ON). Under these conditions, SKPs grow in suspension as spheres. For passaging, SKPs spheres were isolated from the culture medium by centrifugation (5 min, 1200 rpm) and dissociated into single cells by digestion with collagenase XI. For all experiments, passage 3 SKPs were used.

### Gel Contraction Assay

SKPs were cultured in DMEM/F12/Glutamax +10% FBS for three weeks to induce differentiation to SMC. Cells were harvested by trypsinization and contraction of collagen I gels was analyzed using a modification of previously described method [30]. 12-well culture plates were coated with 1% agarose/PBS. Collagen I (Elastin Products Co. Inc., Owensville, Missouri) was diluted in PBS to obtain 3 µg/ml on ice. 800 µl of diluted collagen I was mixed with 100 µl 0.1M NaOH and 100 µl 10× PBS on ice. 300 µl of this mixture was added to each culture well and allowed to polymerize at 37°C for 45 min. Differentiated cells were plated on top of the collagen gels at a cell concentration of 5×10^5^ cells/culture well. Undifferentiated SKPs that had been maintained in EGF/FGF containing culture medium were added to separate collagen gels and used as controls. After 24 hrs culture at 37°C 5% CO_2_, gels were released from the plastic well using a spatula and returned to the cell culture incubator. 8 hrs later, gels were photographed using a Kodak EDAS 290 camera. Image J was used to quantify gel area after contraction.

### Immunofluorescent Staining

SKPs were plated on chamber slides in either EGF/FGF or 3–10% FBS containing DMEM/F12. Rapamycin (Sigma-Aldrich, St.Louis, MO) and/or FGF2 were added at different concentrations directly to the medium. After 20 min or 6 days, cells were fixed in 4% paraformaldehyde/PBS. Fixed cells were permeabilized with 0.1% Triton X/PBS for 5 min at RT. Non specific binding sites were blocked by 1 hr incubation with 3% BSA/PBS at RT. All primary antibodies (Cy3 coupled anti alpha SMactin: Sigma-Aldrich, St.Louis, MO, anti phospho S6: Cell Signaling) were used at a 1∶200 dilution in 1% BSA/PBS over night at 4°C. Primary antibodies were removed by 3×5 min washes in PBS. Secondary Cy2 or Cy3 coupled antibodies (Jackson ImmunoResearch Laboratories, Inc., West Grove, PA) were used at a 1∶200 dilution in 1% BSA/PBS and incubated for 1 hr at RT. Secondary antibodies were removed by 3×5 min washes in PBS. Nuclei were visualized by incubation for 5 min with DAPI/PBS. Slides were mounted with Dako fluorescent mounting medium (Dako, Burlington, ON). Images were taken using either a Zeiss Axiovert 100M inverted fluorescent confocal microscope and LSM510 software or a Leica DMIRE2 inverted fluorescence microscope equipped with a Hamamatsu C9100-12 back-thinned EM-CCD camera and Yokogawa CSU 10 spinning disk confocal scan head and Volocity software. Fluorescence was quantified using Image J or Volocity software.

### Co-culture Experiments

SKPs were isolated as described above from transgenic rats expressing a green fluorescent protein (GFP) under the control of the ubiquitously expressed CAG promoter, which is a combination of the cytomegalovirus early enhancer element, and the chicken beta-actin promoter [31]. For co-culture experiments using bladder organoids, rat bladders were first distended (stretched) using pressure-controlled manometry *ex vivo* for 2 hrs as previously described [28]. Non-stretched *ex vivo* bladders were used as controls. *Ex vivo* bladders were cut into small pieces (organoids) using razor blades and then partially digested in *Clostridium Histolyticum* Collagenase/PBS (1 mg/ml) (Sigma-Aldrich, St.Louis, MO) for 20 min. Collagenase was removed by centrifugation and by PBS washes. GFP positive SKPs were mixed with bladder organoids 1∶1 and cultured in DMEM/F12+3% FBS+antibiotics/antimycotics for 7 days. Alpha SMactin expression was analyzed by immunofluorescent staining as described above. SKPs were identified by staining with an anti GFP specific antibody (Abcam Inc. Toronto, ON).

### Conditioned Medium Experiments

Stretched and unstretched control bladders were isolated, and cultured *ex vivo* as described above [28]. Medium conditioned by *ex vivo* cultured bladders was filtered through 22 µm filter and then diluted 1∶3 with DMEM/F12+10% FBS+antibiotics/antimycotics. SKPs were cultured in conditioned medium for 7 days before expression of smooth muscle specific markers was analyzed by qPCR and IF staining.

### Real Time PCR

RNA from differentiated or non differentiated SKPs was isolated using TRIZOL and10 µg total RNA were reverse transcribed using Superscript II reverse transcriptase (Invitrogen Life Technologies Inc., Burlington, ON) following manufacturer’s instructions. Reverse transcription was initiated using OligodT as primer (Invitrogen Life Technologies Inc., Burlington, ON). Reverse transcription products were diluted 1∶5 with DNase RNase free water, and 2 µl of diluted cDNA was used as template for real time PCR reaction. 2× Dynamo SYBR-green PCR mix (Finnzymes, New England Biolabs, Ipswich, MA) was used following manufacturer’s instructions except final reaction volume was reduced to 25 µl/reaction. C-DNA was amplified using a Peltier Thermal Cycler-200 (MJ Research). Relative transcript levels were analyzed by calculating deltaC(t) values. Amplification of the house keeping gene GAPDH was used to normalize cDNA input. Specific primers were designed using primer 3 software and annealing temperature was optimized by testing amplification efficiency using an annealing temperature gradient between 55–65°C.

Smoothelin AB forward: GGCTCGTCCACTCCAATG.

Smoothelin AB reverse: GGATGAGGAAGAGGAAGAGG.

Smoothelin B forward: TCGGAGTGCTGGTGAATAC.

Smoothelin B reverse: CCCTGTTTCTCTTCCTCTGG.

SMactin forward: GATCACCATCGGGAATGAACGC.

SMactin reverse: CTTAGAAGCATTTGCGGTGGAC.

GAPDH forward: GATCGTGGAAGGGCTAATGA.

GAPDH reverse: GAGCTCTGGGATGACTTTGC.

Sm22 forward: CACCTATCCTCAGCCTCAGC.

Sm22 reverse: TTCAAAGGACATTGGCTTCC.

Cnn1 forward: ACAACACCCAAAGGAAGCAC.

Cnn1 reverse: TCACTGCAAAACCAAACTGC.

Myh11 forward: CTACTCGGGCCTCTTCTGTG.

Myh11 reverse: TTCCAGCTCCAGACTCACCT.

Myocd forward: ACTGAGGTGAGCCTCTCCAA.

Myocd reverse: GCTTCCCAGAGTCTGACTGG.

### Statistical Analysis

Statistical significance of results was determined by ANOVA and Student’s ttest. A P value of <0.05 was considered significant.

## Results

### SKPs Differentiate in vitro into Contractile Smooth Muscle Like Cells

Recently, Steinbach et al. [21] demonstrated that rat SKPs differentiate into cells resembling vascular SMC when exposed to FBS. Since skin is a relatively accessible cell source for tissue engineering/regeneration purposes, we decided to evaluate whether adult SKPs would be useful as source of visceral SMC for bladder regeneration approaches. Initially, we confirmed that FBS induces *in vitro* smooth muscle differentiation of SKPs that had been isolated from adult rat skin. As described previously [15], [16], SKPs cultured as non-adherent spheres in EGF/FGF containing medium maintain their multipotency. To induce smooth muscle differentiation, SKPs were cultured in FBS (3, 10 or 15%) containing medium for one to three weeks and expression of smooth muscle specific markers was analyzed by qPCR and immunofluorescent (IF) staining. Whereas the majority of cells cultured in EGF and FGF containing medium continued to grow as non-adherent, multi-cellular spheres, the majority of cells cultured in FBS (15%) containing medium adhered to tissue culture plastic after seven days. Real time PCR revealed a strong and significant up regulation of the smooth muscle markers alpha SMactin, calponin, SM22, myocardin, and myosin heavy chain (Figure S1) in response to FBS treatment. To investigate differentiation of individual cells in more detail and to determine the percentage of cells undergoing differentiation, we stained SKPs undergoing differentiation with alpha SMactin specific antibodies. Staining was heterogeneous (data not shown) with about 50% of cells displaying strong expression of alpha SMactin and the remaining cells displaying only weak or no expression of alpha SMactin. To test if FBS differentiated cells are contractile, we employed gel contraction assays. Gel contraction was significantly increased (31% P<0.05) when SKPs were differentiated for three weeks in FBS containing medium compared to undifferentiated controls (Figure S2). These results confirmed that *in vitro,* SKPs isolated from adult rat skin can differentiate into functional, smooth muscle like cells.

### Bladder Strain-induced Microenvironment Attenuates SMC Differentiation of SKPs

Since SKPs can be induced to differentiate along a smooth muscle lineage, these cells might be an excellent source of visceral SMC for use in bladder regeneration. Bladder outlet obstruction, one pathologic situation necessitating bladder regeneration creates a microenvironment that induces bladder SMC phenopathology characterized by hyperproliferation and de-differentiation [28], [29], [32]. To investigate the effect of this pathologic bladder microenvironment on SKP differentiation, to more specifically test how the bladder strain-induced microenvironment regulates the fate determination of SKPs, we employed a combination of *ex vivo* organ culture and bladder derived organoid-SKPs co-culture.

**Figure 1:**
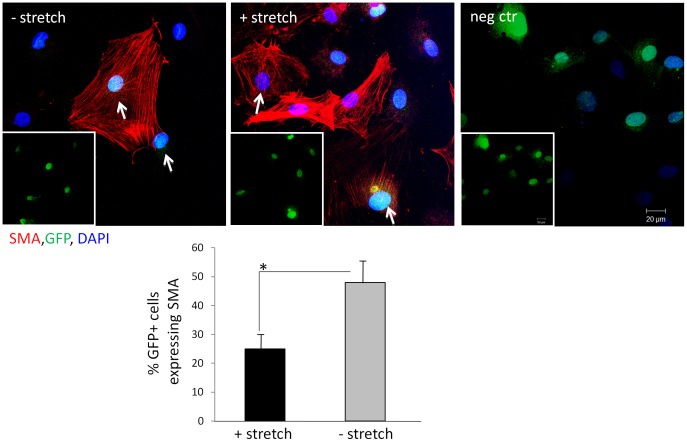
The microenvironment of stretch injured adult bladders attenuates SMC differentiation of SKPs. GFP+ SKPs were co-cultured with organoids isolated from stretched or non-stretched adult rat bladders. After one week, SMA expression was analyzed by IF staining and the percentage of GFP+/SMA+ cells was quantified. Inserts show GFP+ cells. Arrows indicate GFP+/SMA+ cells. Graph shows mean +/− SE of n = 100 cells.

We previously developed an organ culture protocol that allows us to stretch intact bladders in a controlled fashion *ex vivo*. SKPs were co-cultured with organoids isolated from either *ex vivo* stretched (2 hrs) or non-stretched bladders and alpha SMactin expression was analyzed by IF after one week (Figure 1). SKPs that were used for this experiment expressed GFP *in* *vitro* and during differentiation and could therefore be distinguished from non-GFP bladder organoid cells. As observed previously, SKPs cultured in FBS containing medium expressed alpha SMactin. Interestingly, the number of SKPs expressing alpha SMactin was significantly reduced (23% vs. 49% P<0.05) when SKPs were co-cultured with organoids that had been isolated from *ex vivo* stretched bladders compared to SKPs co-cultured with organoids that had been isolated from non-stretched bladders, suggesting that a strain induced bladder microenvironment suppresses differentiation of SKPs toward SMC (Figure 1).

### SMC Differentiation of SKPs is Modified by Bladder Strain Induced Diffusible Factors

Since exposure of SKPs to a strain induced bladder organoid context inhibited their differentiation, we next assessed whether this response may be due to diffusible factors or SKP-cell contact. Previous research in our laboratory determined that bladder strain leads to increased production of growth factors and MMPs by bladder SMC activated by mechanical stretch [28]. The released MMPs degrade bladder extracellular matrix, leading to accumulation of reactive matrix fragments and neoepitopes, which stimulate cell signalling pathways via cell surface receptors. As a consequence, conditioned medium obtained from *ex vivo* stretched bladders may mimic the effects of bladder strain. For example, treatment of non-stretched bladder SMC with stretched-bladder conditioned medium resulted in activation of the ERK1/2 signalling pathway and increased cell proliferation [28].

**Figure 2:**
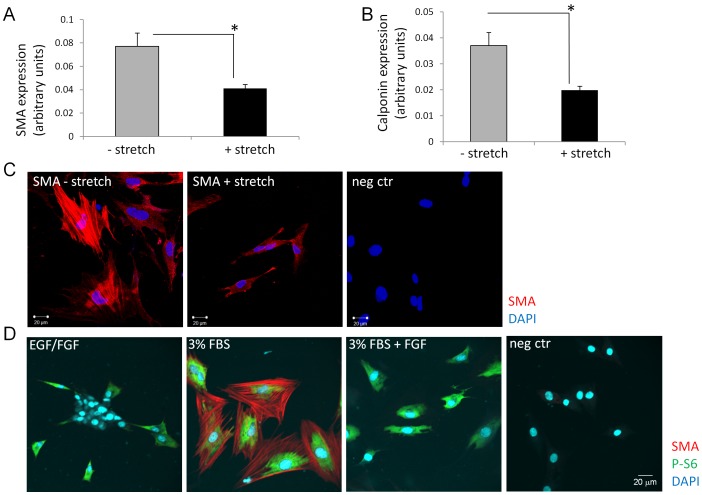
Bladder stretch induced diffusible factors suppress SMC differentiation of SKPs. SKPs were cultured in conditioned medium obtained from *ex vivo* stretched or non-stretched adult bladders. A: SKPs were cultured in medium conditioned by *ex vivo* stretched or non-stretched bladders for one week. SMA expression was analyzed by qPCR (A) and IF (C), Calponin expression was quantified by qPCR (B). Graphs show mean +/− SE of n = 4. Representative images of n = 10 are shown in C. D: SKPs were differentiated in medium containing 3% FBS or 3% FBS +4 ng/ml FGF2/bFGF for one week. SKPs cultured in medium containing EGF and bFGF were used as control. SMA expression was analyzed by IF staining. Representative images of n = 10 are shown.

To investigate whether diffusible factors are responsible for the inhibitory effect of bladder stain on SKPs differentiation, we cultured SKPs in conditioned medium obtained from *ex vivo* stretched or non-stretched bladders (Figure 2). QPCR revealed that alpha SMactin (Figure 2 A) and calponin (Figure 2 B) expression was significantly reduced (1.75 fold P<0.05 for SMA and Calponin) in SKPs that had been exposed to conditioned medium from stretched bladders compared to SKPs cultured in conditioned medium from bladders that had not been stretched. Alpha SMactin IF also revealed a reduction in alpha SMactin expression, coordinate with the qPCR results (Figure 2 C). Taken together, these data suggested that diffusible factors released into the strain induced bladder microenvironment are sufficient to cause suppression of SKP differentiation.

Smoothelin, a SMC specific cytoskeleton protein, is expressed as two isoforms, a full length protein, primarily expressed by vascular SMCs and a truncated protein, primarily expressed by visceral SMCs. Relative expression of these two isoforms has previously been used to distinguish between vascular and visceral SMCs [21]. Since sequence of the smaller visceral isoform is also present in the full length vascular isoform, it is not possible to design primers that only amplify the visceral isoform. In contrast, primers that specifically amplify the vascular isoform can be designed and used to determine the relative amount of vascular smoothelin to overall smoothelin [21]. Indeed, qPCR using the same amplification strategy revealed that exposure of SKPs to conditioned medium from *ex vivo* stretched bladders increases expression of the vascular SMC specific B isoform of smoothelin, compared to undifferentiated SKPs or SKPs exposed to conditioned medium isolated from non-stretched bladder cultures (Figure S3), which is consistent with suppressing differentiation toward a visceral smooth muscle phenotype.

Previous experiments in our laboratory identified MMP activity as one of the factors responsible for bladder strain induced SMC proliferation and de-differentiation [28]. Conditioned medium obtained from *ex vivo* cultures of strained bladders induced hyperplasia and de-differentiation of mature cultured SMC *in vitro.* However, SMC differentiation was maintained in conditioned medium when bladders underwent stretch in presence of doxycycline, a broad-range MMP inhibitor [33], [34], [35], [36]. To test whether MMPs are required for the observed inhibition of SMC differentiation of SKPs, we exposed SKPs to conditioned medium obtained from bladders that underwent stretch in presence of doxycycline. SMC differentiation of SKPs was quantified by IF staining for alpha SMactin (Figure S4). Interestingly, conditioned medium obtained from doxycycline treated *ex vivo* distended bladders further decreased, rather than rescued alpha SMactin expression of SKPs compared to conditioned medium obtained from bladders distended without doxycycline.

Bladder obstruction has also been shown to increase expression of growth factor FGF2/bFGF by bladder SMCs and uroepithelial cells, and FGF2/bFGF has previously been shown to increase proliferation of bladder SMCs, making it a candidate responsible for the observed inhibitory effect of bladder strain on SMC differentiation of SKPs [37], [38], [39], [40]. To test whether FGF2/bFGF indeed counteracts SMC differentiation of SKPs, we exposed SKPs undergoing SMC differentiation to different concentrations (4 ng–100 ng/ml) of FGF2/bFGF and quantified alpha SMactin expression by IF staining (Figure 2 D). Even at the lowest (4 ng/ml) concentration, FGF2/bFGF strongly suppressed alpha SMactin expression (8.8 fold P<0.05) compared to FBS treated SKPs, suggesting that FGF2/bFGF is one of the bladder strain induced factors that are responsible for decreased SMC differentiation of SKPs.

### SMC Differentiation of SKPs is Regulated by mTOR

**Figure 3:**
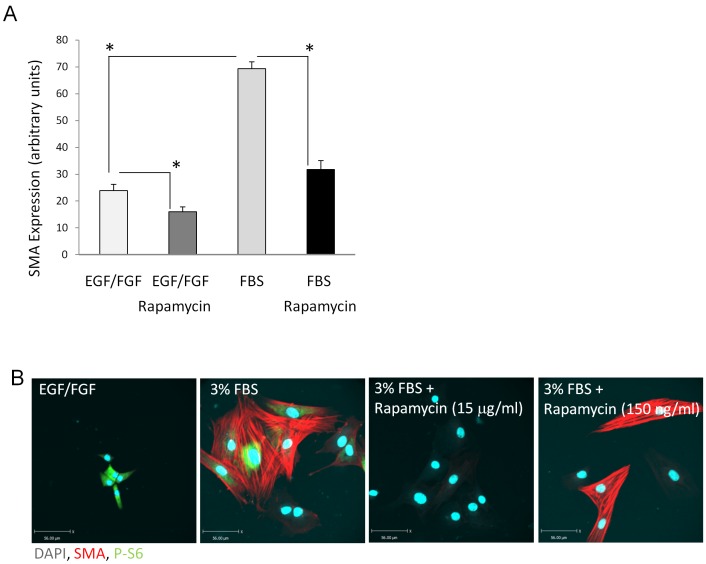
Rapamycin treatment down regulates SMA expression of SKPs. SKPs were cultured in medium containing either EGF/FGF +/− Rapamycin or 15% FBS +/− Rapamycin for one week. A: SMA expression was quantified by qPCR. Graph shows mean +/− SE of n = 4. B: SKPs were cultured in medium containing either EGF/FGF +/− Rapamycin or 3% FBS +/− Rapamycin for one week. SMA expression and S6 phosphorylation was analyzed by IF staining. Representative images of n = 10 are shown.

**Figure 4:**
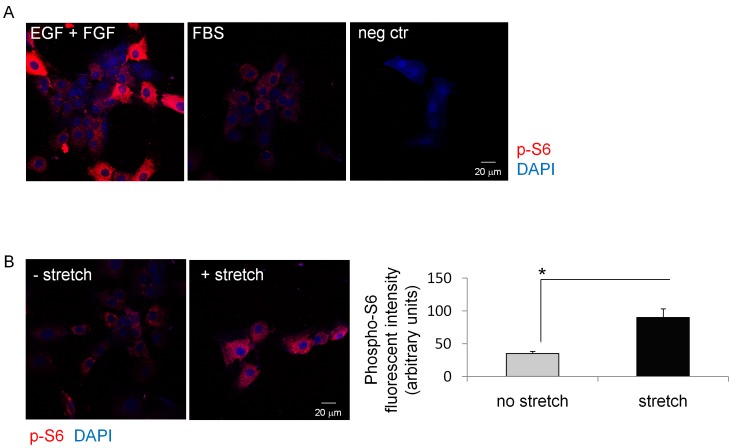
SMC differentiation of SKPs correlates with reduced S6 phosphorylation. A: SKPs were cultured in medium containing 15% FBS for 20 min. S6 phosphorylation was analyzed by IF staining. Representative images on n = 10 are shown. B: SKPs were exposed to conditioned medium from either stretched or non-stretched bladders for 20 min. S6 phosphorylation was analyzed by IF staining. Fluorescent intensity was quantified by image analysis using Volocity software. Graph represents mean +/− SE of n = 50 cells.

We recently demonstrated that the mammalian target of rapamycin (mTOR) signalling cascade is activated in bladder SMC exposed to strain, denatured collagen and/or hypoxia, three canonical stimuli that play key roles in pathobiology of bladder obstruction [29]. Inhibition of mTOR with rapamycin reduces the visceral SMC proliferation and de-differentiation induced by strain, denatured collagen, or hypoxia *in vitro* [29]. To investigate whether rapamycin had a similar effect on smooth muscle differentiation of SKPs, we differentiated SKPs in the presence of 150 ng–15 µg/ml rapamycin or DMSO vehicle for 7 days and analyzed expression of SMC specific genes by qPCR (Figure 3 A, Figure S5). 15 µg/ml rapamycin significantly (P<0.05) decreased expression of alpha SMactin, Myocardin, Calponin, Myh11 and Sm22 whereas DMSO control had no effect. Cell viability was not affected by rapamycin treatment. The effect of rapamycin on SMA expression was then analyzed on a single cell level by alpha SMactin IF staining (Figure 3 B). As demonstrated by qPCR, 15 µg/ml rapamycin reduced alpha SMactin expression by SKPs compared to SKPs that were undergoing differentiation without rapamycin. Treatment with low concentrations of rapamycin (150 ng/ml, Figure 3 B) had no effect on alpha SMactin expression. We then used phosphorylation of the ribosomal protein S6 (P-S6), a downstream target of mTOR as readout for activation of the mTOR pathway. We first analyzed S6 phosphorylation in SKPs exposed to 10% FBS by IF staining and compared it to undifferentiated cells cultured in EGF/FGF containing medium (Figure 4 A). S6 phosporylation was heterogeneous in undifferentiated cells with 30% of cells, mainly located at the edge of cell groups, staining strongly with a P-S6 specific antibody. 20 min treatment with 10% FBS containing medium resulted in an overall reduction of S6 phosphorylation, all cells displayed low IF staining, demonstrating a coordinate down regulation of mTOR activity with induction of SMC differentiation of SKPs.

Treatment of SKPs with conditioned medium from *ex vivo* stretched bladders reduced SMC differentiation of SKPs compared with SKPs that were treated with conditioned medium from non-stretched bladders. To test whether this inhibitory effect of conditioned medium from stretched bladders also affects S6 phosphorylation, SKPs were treated with conditioned medium from stretched or non-stretched bladders for 20 min and S6 phosphorylation was analyzed by IF staining (Figure 4 B). S6 phosphorylation was increased in SKPs treated with conditioned medium obtained from *ex vivo* stretched bladders compared to SKPs treated with conditioned medium obtained from non-stretched bladders (2.3 fold P<0.05). Furthermore, S6 phosphorylation was increased in SKPs treated with conditioned medium from stretched bladders plus doxycycline compared to SKPs treated with conditioned medium from stretched bladders minus doxycycline (Figure S4). Combined, these results suggested that increased S6 phosphorylation and therefore mTOR activity may coordinately regulate the suppression of SMC differentiation of SKPs. Interestingly, although all tested concentrations of rapamycin (150 ng–15 µg) inhibited S6 phosphorylation, only high rapamycin concentrations (15 µg/ml) inhibited SMA expression (Figure 4C). These results suggested that S6 phosphorylation itself is not a necessary condition for the mTOR-mediated control of SMC differentiation of SKPs.

**Figure 5:**
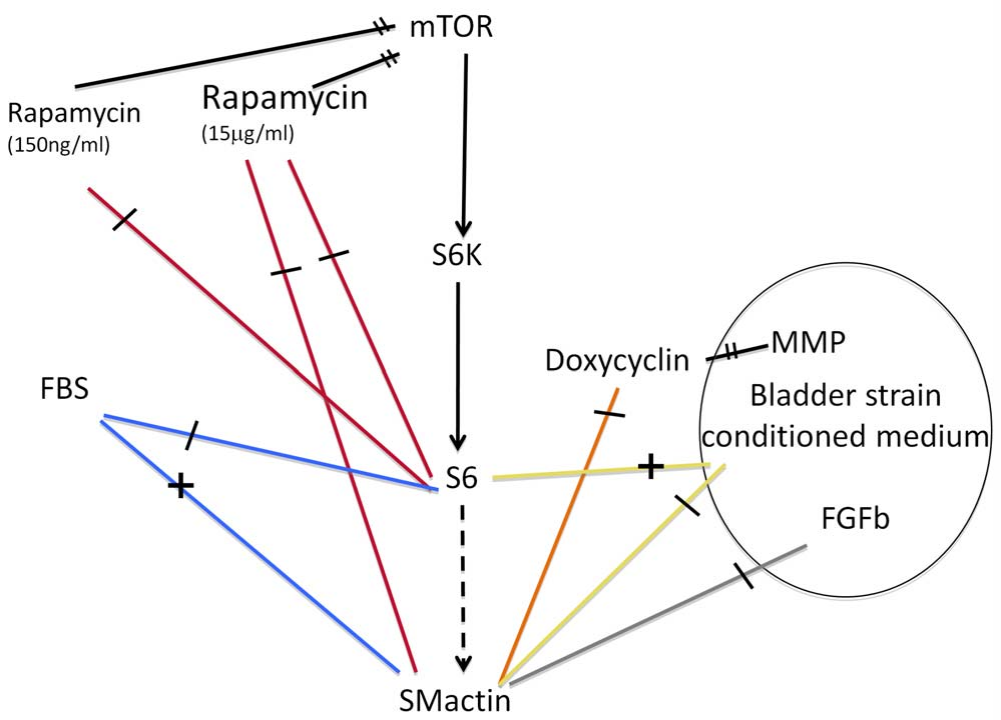
Overview of described effects on SMA expression and S6 phosphorylation. Graphic depicts elements of the mTOR pathway and effects of FBS, conditioned medium, FGFb, doxycycline and rapamycin on S6 phosphorylation and alpha SMactin expression. MMPs and FGFb are putative components of conditioned medium isolated from *ex vivo* stretched bladder cultures. Overview of described effects on SMA expression and S6 phosphorylation: FBS treatment (blue) reduces S6 phosphorylation and increases SMA expression. In contrast, conditioned medium harvested from *ex vivo* stretched bladders increases S6 phosphorylation and decreases SMA expression (yellow) relative to FBS treatment. MMPs (orange) and FGFb (light gray), two putative components of conditioned medium harvested from *ex vivo* stretched bladders both decrease SMA expression of SKPs. Effect of rapamycin (red) on SMA expression is concentration dependent. High concentration of rapamycin inhibit SMA expression, low concentration of rapamycin had no effect on SMA expression.

Collectively, we have shown that bladder strain induced microenvironmental factors attenuate SMC differentiation of SKPs (Figure 5). In contrast to its effect on mature differentiated visceral SMC, rapamycin treatment reduced SMC differentiation potential of undifferentiated progenitor cells.

## Discussion

Although progress has been made in stem cell biology research, routine and predictable programming of progenitor cells for tissue regeneration remains elusive. A principle challenge is inefficient *in vivo* integration/survival and differentiation of progenitor cells. Any use of progenitor cells *in vivo* will result in their exposure to an abnormal microenvironment that differs from normal intact tissue, whether it is the pathological microenvironment of the original organ requiring regeneration or the nascent microenvironment of a regenerating tissue. Recent research has demonstrated that a pathological tissue microenvironment modifies cell behaviour and that these effects are persistent and irreversible. For example, bladder hypertrophy and cell hyperplasia induced by bladder obstruction often persists well beyond surgical removal of the obstruction. Also, culture of primary visceral SMC on denatured collagen, mimicking matrix damage induced by bladder obstruction, stimulates cell proliferation compared to culture on native collagen. Importantly, the increased proliferation persists even after cells from denatured collagen are passaged onto native collagen [32]. These observations stress the importance of the microenvironment in disease pathobiology and its effect on cell phenotype. As a consequence, tissue regeneration/engineering strategies using progenitor cells have to consider the specific microenvironment of the pathologic tissue.

Recently, SKPs have been shown to differentiate into smooth muscle like cells using FBS as differentiation inducing factor [21]. SKPs differentiated by FBS express a variety of smooth muscle specific genes such as alpha SMactin, myocardin and calponin and are contractile as demonstrated by collagen gel contraction assays. We investigated the effect of a bladder strain-induced microenvironment on smooth muscle differentiation of SKPs. Co-culture of SKPs with bladder cells isolated from bladders that had undergone stretch in an *ex vivo* organ culture system resulted in down regulation of smooth muscle specific gene expression in SKPs. Furthermore, exposure of SKPs to culture medium that first had been conditioned by *ex vivo* culture of stretched bladders also significantly reduced expression of smooth muscle specific genes in SKPs.

Many of the genes that are being assessed to determine visceral smooth muscle specific cell differentiation are also expressed by vascular as well as visceral SMC and therefore cannot be used to distinguish between these two different smooth muscle subtypes. However, the smooth muscle specific protein smoothelin, a cytoskeletal protein, is expressed as two isoforms [41], [42], [43] which are the result of alternative promoter use [44]. Vascular SMC primarily express the longer smoothelin B isoform whereas bladder SMC expresses primarily the shorter A isoform. The isoform A specific promoter is regulated by myocardin and serum response factor which also regulates alpha SMactin and desmin [45] expression. Steinbach et al used relative expression of these two smoothelin isoforms to determine whether SKPs can be differentiated into vascular or visceral SMC and demonstrated that SKPs differentiated by exposure to FBS primarily express smoothelin B, therefore resembling vascular SMC [21].

SKP exposure to conditioned medium provides further insight into this specific SMC determinancy. Conditioned medium prepared from either stretched or non-stretched *ex vivo* bladders did not significantly influence overall smoothelin expression but, importantly, modified the balance between the A and B isoforms. Conditioned medium from stretched bladders strongly increased expression of the smoothelin B (vascular>visceral) isoform, whereas conditioned medium from non-stretched bladders decreased expression of this blood vessel specific isoform (vascular<visceral), possibly increasing visceral SMC phenotype observed in differentiated SKPs. Although differentiation into vascular SMC may support or increase neoangiogenesis, more importantly these observations underscore the need to understand how the microenvironment may counter-orchestrate progenitor cell differentiation in otherwise unpredictable ways.

Stretch of bladder SMC activates cell signalling pathways resulting in production and release of growth factors such as HB-EGF and bFGF [37], [38], [39], [40], [46], [47], both of which have been correlated with bladder SMC proliferation. Furthermore, we previously demonstrated that bladder stretch increases activity of MMP 7 (matrilysin) as well as the gelatinases MMP 2 and 9 activity in bladder tissue [28], [48]. The increased MMP activity leads to remodelling of the bladder strain induced microenvironment in part by degradation of extracellular matrix resulting in the release of matrix fragments or neoepitopes that act as signalling mediators. This ECM remodelling also releases growth factors whose concentrations are regulated by ECM sequestration. Moreover, MMP activity is also required for activation of protein signalling mediators. Indeed, mechanical stretch activates ERK1/2 in *ex vivo* bladder cultures resulting in the release of MMP into the medium where this conditioned medium can then induce ERK1/2 activation in non-stretched visceral cells [28]. Treatment with MMP inhibitors during bladder stretch inhibits the ERK stimulating activity of conditioned medium. In the present study, conditioned medium harvested from stretched bladders pre-treated with the broad-spectrum MMP inhibitor doxycycline, further suppresses smooth muscle differentiation of SKPs even beyond that decreased by medium conditioned without doxycycline. Doxycycline is known to block activity of a variety of MMPs including MMP-1, -8, -7, and -13 [33], [34], [35], [36]. Given the multi-functional role of the protease function of MMP’s to activate growth factors, participate in protein complexes, as well as their editing and remodelling function of extracellular matrix proteins, enhanced de-differentiation while inhibiting MMP activity suggests that MMP activity may participate in a balancing or regulating SKP determinacy within these specific functional contexts. This role for MMPs in supporting SMC differentiation of SKPs is likely not mediated through ERK since ERK inhibition was observed to increase SMC differentiation (data not shown).

Another factor released in response to bladder strain is the growth factor FGF2 or bFGF [37], [38], [39], [40]. Combined with EGF, this growth factor prevents differentiation and maintains pluripotency of SKPs. We therefore tested the effect of FGF2 on FBS induced smooth muscle differentiation of SKPs. Even relatively low concentrations of FGF2 strongly inhibit alpha SMactin expression by SKPs, suggesting that FGF2 is one of the factors responsible for the negative effect of bladder strain on SMC differentiation of SKPs.

Bladder stretch also strongly activates the mTOR pathway and inhibition of this pathway with the mTOR inhibitor rapamycin reduces strain induced cell proliferation and visceral SMC de-differentiation [29]. The mTOR pathway is an important regulator of cell proliferation, differentiation and protein translation and is a sensor of nutrient and energy levels. Moreover, mTOR activity has recently been associated with progenitor cell differentiation. For example, mTOR activity is required to maintain pluripotency of human embryonic stem cells [49], [50] and inhibition of mTOR signalling by low concentrations of rapamycin induces SMC differentiation of mesenchymal stem cells [51]. These observations prompted us to investigate the role of mTOR pathway in visceral SMC differentiation of SKPs, a progenitor cell with a relatively narrower differentiation repertoire than pluripotent embryonic stem cells. Phosphorylation of S6, a downstream target of mTOR, was higher in SKPs with restrained SMC differentiation following treatment with conditioned medium from stretched bladders compared to cells treated with conditioned medium from non-stretched bladders. Furthermore, S6 phosphorylation was also increased in SKPs exposed to conditioned medium from stretched bladders treated with MMP inhibitors (vs. without MMP inhibitors), whereas the differentiation conditions of FBS treatment blunted S6 phosphorylation. These results suggest that increased S6 activation is associated with decreased SMC differentiation of SKPs, which is consistent with observation made in human embryonic stem cells [49], [50].

These data then raised the question of how mTOR inhibition would modify SMC differentiation of SKPs. While treatment with low concentration of rapamycin had no significant effect, treatment with high concentrations inhibited SMC differentiation of SKPs. Thus, FBS blunts the downstream mTOR component phospho S6 and *initiates* SKP differentiation, while, conversely, directly inhibiting mTOR with rapamycin *inhibits* SKP differentiation. Interestingly, although low rapamycin concentrations also inhibit S6 phosphorylation, SMC differentiation of SKPs is not blocked until high concentrations of rapamycin are used. These results highlight a tight regulation of SKP differentiation through the mTOR cascade: SM differentiation of SKPs requires an activity of the mTOR pathway that is neither too high nor too low. Alternatively, requirements for mTOR pathway activity may differ between early vs. late stages of differentiation, or by discrete activities of each component, mTOR or phospho S6, on differentiation. Upstream PI3K/Akt activation also induces S6 phosphorylation raising the possibility that activation of Akt influences SMC differentiation of SKPS. Furthermore, the effect of low rapamycin concentrations on inhibition of S6 phosphorylation despite progression towards differentiation may suggest that differentiation may also involve an mTOR/S6 independent mechanism. High concentrations of rapamycin and/or prolonged treatment with rapamycin have been reported to inhibit mTORC2 in addition to mTORC1, raising the possibility that mTORC2 is involved in smooth muscle differentiation of SKPs [52], [53].

In conclusion, we have developed a combination of *ex vivo* and *in vitro* strategies allowing molecular characterization of progenitor cell differentiation exposed to a pathologic microenvironment, based at least in part on the mTOR signaling cascade. Using this approach, we demonstrate that an organ specific, and physiologically anticipated mechanical strain-induced microenvironment has a paradoxical effect on preferred SMC differentiation of SKPs by modifying a tightly regulated key signalling pathway. Our results demonstrate that fine tuning of signalling pathway activity influenced by microenvironmental cues needs to be an important consideration in tissue regeneration studies employing exogenous precursor cells.

## Supporting Information

Figure S1 **FBS treatment induces smooth muscle marker expression in SKPs.** SKPs were cultured in either EGF/FGF or 15% FBS containing medium for 1 week. Expression of SMC marker was quantified by qPCR. Graphs show mean +/− SE of n = 4.
(TIF)

Figure S2 **FBS differentiated SKPs are contractile.** SKPs were differentiated for 3 weeks in FBS (15%) containing medium. Differentiated SKPs were plated on Collagen 1 gels and gel contraction was quantified after 24 hrs. Undifferentiated SKPs or cell free gels were used as controls. Graph shows mean +/− SE of n = 4.
(TIF)

Figure S3 **Conditioned medium from stretched bladders increases Smoothelin B expression.** SKPs were treated with conditioned medium from *ex vivo* stretched or non stretched bladders for one week. Expression of Smoothelin A+B and Smoothelin B was quantified by qPCR using primer that either amplify both Smoothelin isoforms or only Smoothelin B. Expression was standardized using amplification of GAPDH. Relative expression was quantified as delta delta Ct. Graph represent mean +/− SE of n = 4.
(TIF)

Figure S4 **Doxycycline modifies SMA expression and S6 phosphorylation in SKPs.** SKPs were exposed to conditioned medium from *ex vivo* stretched bladders +/− Doxycycline. After 20 min, S6 phosphorylation was analyzed by IF staining. Representative images on n = 10 are shown. SMA expression was analyzed after one week by IF staining. Representative images on n = 10 are shown.
(TIF)

Figure S5 **Rapamycin treatment inhibits SMC marker expression in SKPs.** SKPs were cultured in medium containing 15% FBS +/− Rapamycin. SM marker expression was analyzed by qPCR using primer specific for Myocardin, Calponin, Myosin heavy chain or Sm22. Graphs represent mean +/− SE of n = 4.
(TIFF)
