# Supplementary figures and images for: Pathologic Bladder Microenvironment Attenuates Smooth Muscle Differentiation of Skin Derived Precursor Cells: Implications for Tissue Regeneration
Source: PLoS One. 2013 Apr 1;8(4):e59413. doi: 10.1371/journal.pone.0059413 (PMC3613403; doi:10.1371/journal.pone.0059413)

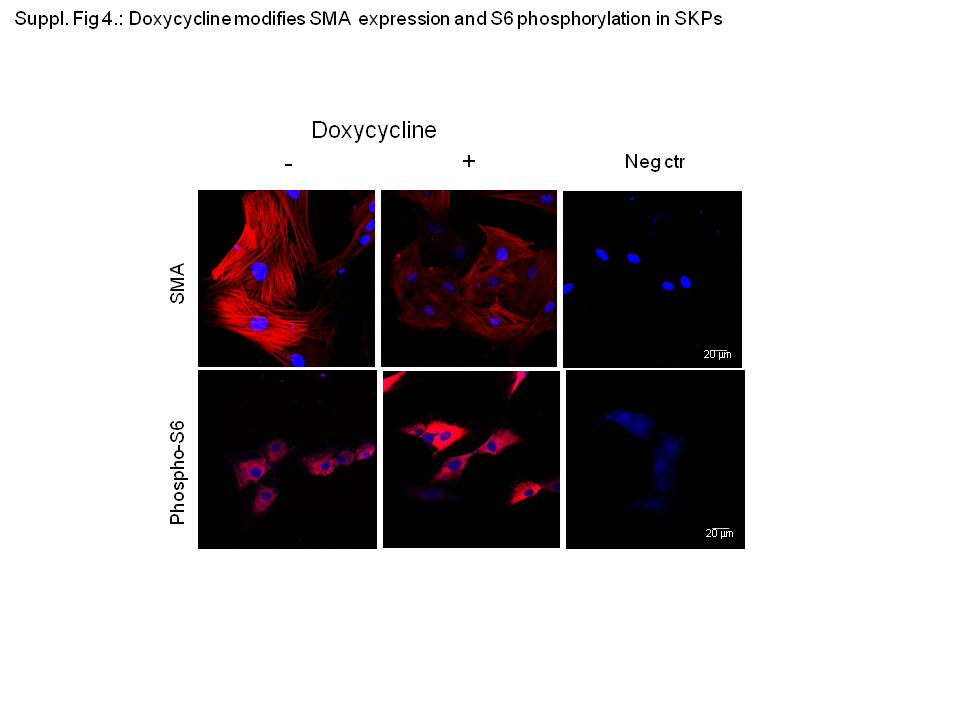

Supplement: Figure S4 — Doxycycline modifies SMA expression and S6 phosphorylation in SKPs. SKPs were exposed to conditioned medium from ex vivo stretched bladders +/− Doxycycline. After 20 min, S6 phosphorylation was analyzed by IF staining. Representative images on n = 10 are shown. SMA expression was analyzed after one week by IF staining. Representative images on n = 10 are shown. (TIF) [file pone.0059413.s004.tif]

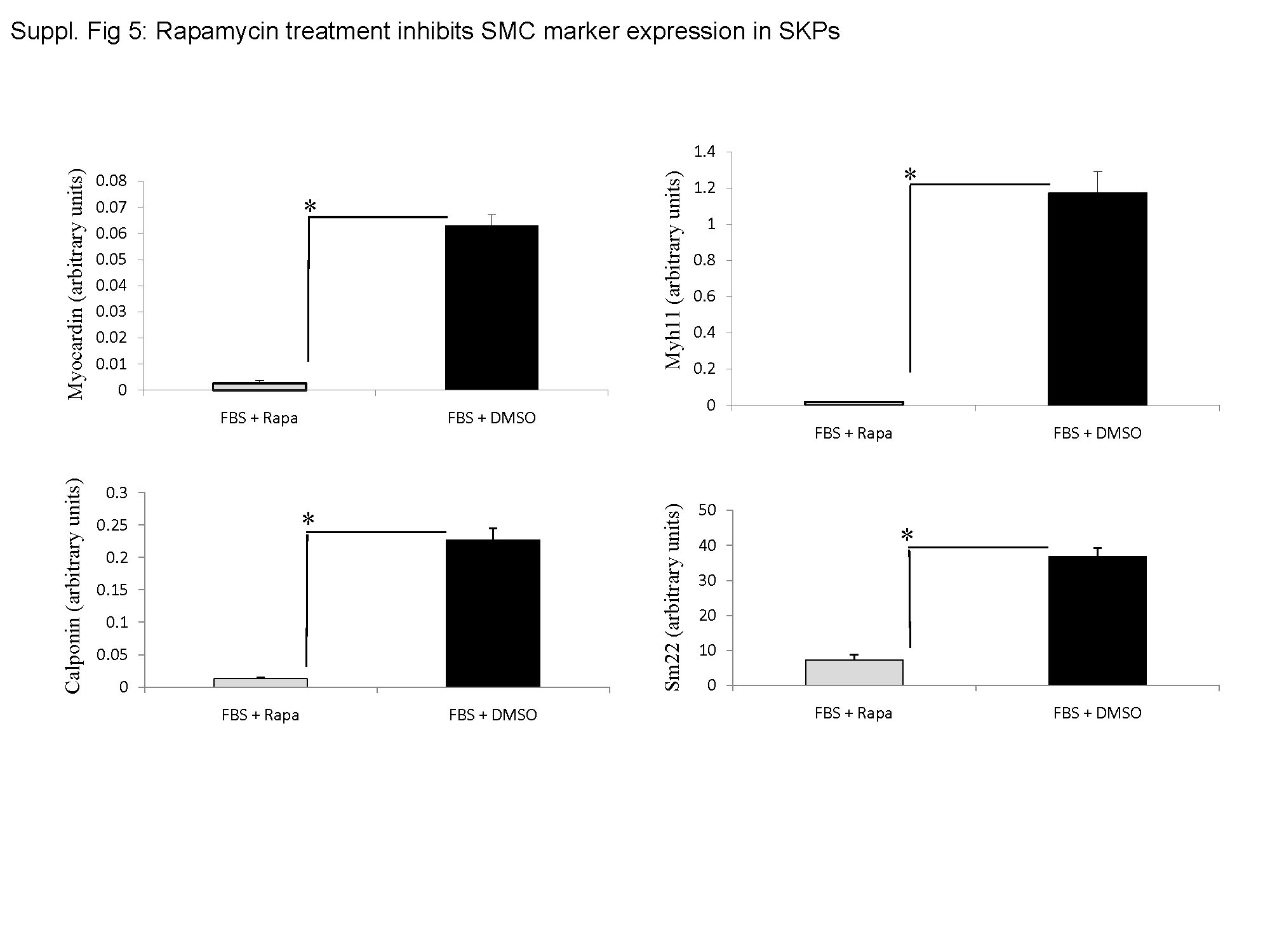

Supplement: Figure S5 — Rapamycin treatment inhibits SMC marker expression in SKPs. SKPs were cultured in medium containing 15% FBS +/− Rapamycin. SM marker expression was analyzed by qPCR using primer specific for Myocardin, Calponin, Myosin heavy chain or Sm22. Graphs represent mean +/− SE of n = 4. (TIFF) [file pone.0059413.s005.tif]
